# Supplementary material for: Assessing the Level of Knowledge, Implementation Practices, and Use of Digital Applications for the Optimal Adoption of the Nutrition Care Process in Greece
Source: Nutrients. 2024 May 31;16(11):1716. doi: 10.3390/nu16111716 (PMC11174944; doi:10.3390/nu16111716)
Supplement: Supplementary file 1 [file nutrients-16-01716-s001.zip › nutrients-3035522-supplementary.pdf]

Supplementary Material

Table S1: Additional demographic characteristics of the participants.

**Variables**	**No Participants who were not aware of the NCP (%)**	**No Participants who were aware of the NCP (%)**	**Total No of Participants (%)**	**p-value**
**Country of Undergraduate Studies**				
Greece	85 (32.1)	180 (67.9)	265 (100.0)	0.407
Cyprus	0 (0.0)	4 (100.0)	4 (100.0)	
Other	2 (20.0)	8 (80.0)	10 (100.0)	
**Place of Residency**				
Aegean Islands	7 (53.8)	6 (46.2)	13 (100.0)	*0.005
Central Greece	24 (20.5)	93 (79.5)	117 (100.0))	
Crete	5 (41.7)	7 (58.3)	12 (100.0)	
Eptanisa	2 (66.7)	1 (33.3)	3 (100.0)	
Epirus	2 (33.3)	4 (66.7)	5 (100.0)	
Macedonia	31 (33.7)	61 (66.3)	92 (100.0)	
Peloponnese	6 (42.9)	9 (57.1)	14 (100.0)	
Thessaly	5 (31.3)	11 (68.8)	16 (100.0)	
Thrace	5 (83.3)	1 (16.7)	6 (100.0)	

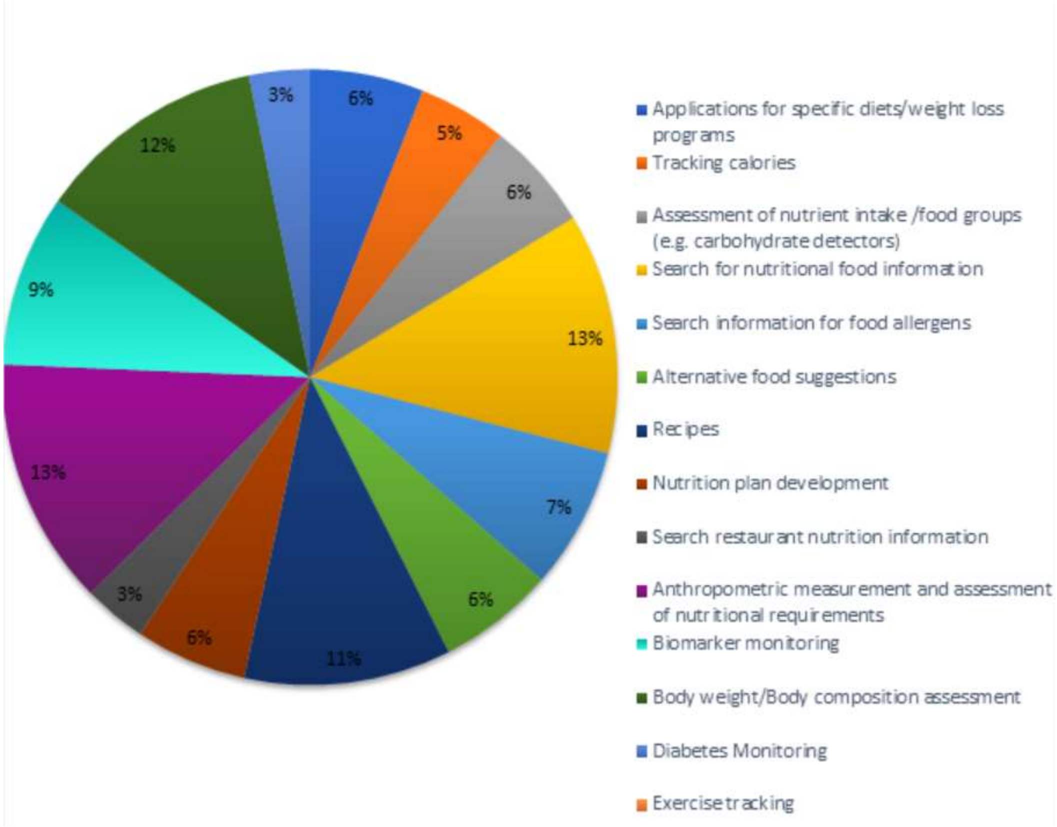

Figure S1. Types of services that dietitians provide with the use of digital applications in dietetic practice.
N = 153

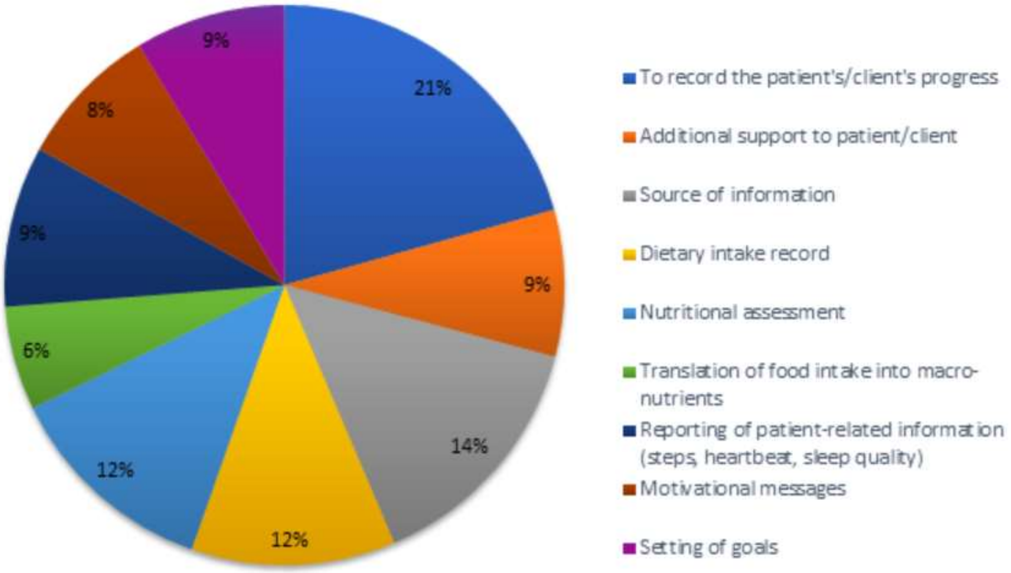

Figure S2. Reasons for using digital tools/platforms during dietetic practice.
N = 153

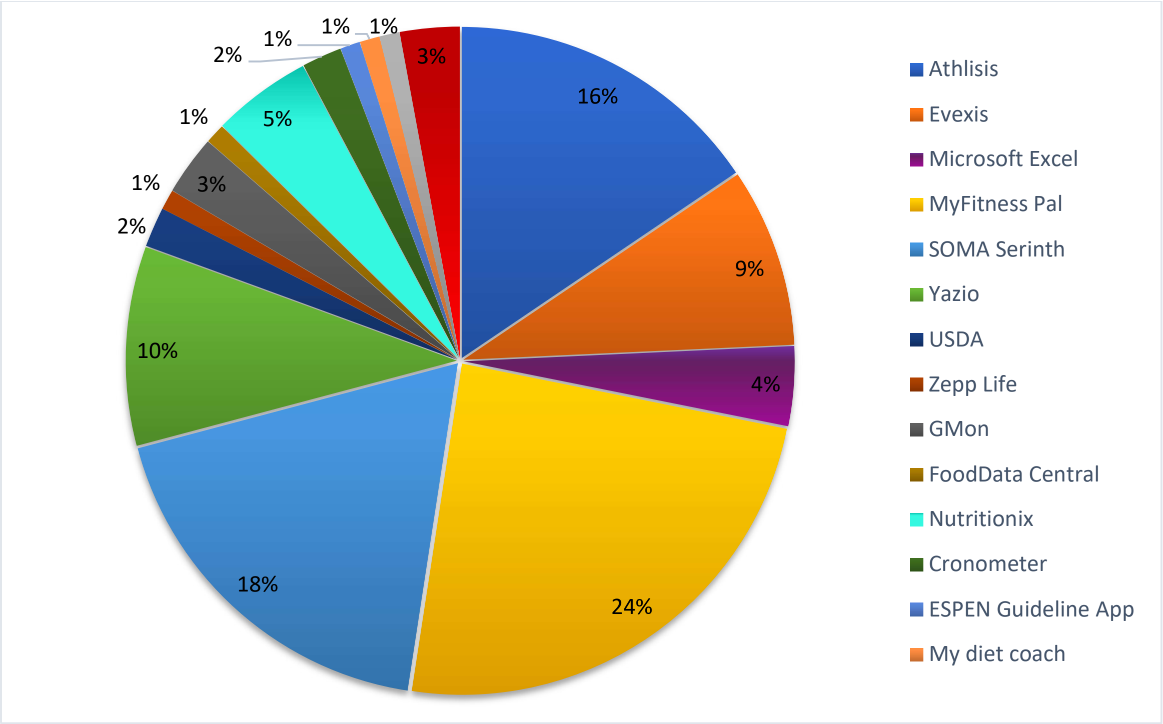

**N = 153*

Figure S3. Digital apps/platforms dietitians use to support dietetic practice.
